# Cardiac Catheterization Procedures in Patients with HIV: A Retrospective Analysis

**DOI:** 10.3390/jcdd8040033

**Published:** 2021-03-27

**Authors:** Bertrand Ebner, Louis Vincent, Jelani Grant, Claudia Martinez

**Affiliations:** 1 Department of Internal Medicine, Jackson Health System/University of Miami Miller School of Medicine, Miami, FL 33136, USA; louis.vincent@jhsmiami.org (L.V.); jelani.grant@jhsmiami.org (J.G.); 2 Department of Cardiovascular Disease, University of Miami Miller School of Medicine, Miami, FL 33136, USA; cmartinez5@med.miami.edu

**Keywords:** percutaneous coronary intervention, HIV/AIDS, cardiovascular disease

## Abstract

With the advent of effective antiretroviral therapies, there has been a decrease in HIV-related mortality, but an increase in non-AIDS-related comorbidities including cardiovascular disease (CVD). We sought to investigate current status of cardiac catheterization (CC) procedures in people with HIV (PWH). This is a retrospective study done at a University Hospital in South Florida between 2017 and 2019. Medical records from 985 PWH indicated that CC was performed in 1.9% of the cases. Of the PWH who underwent CC, 68% were found to have obstructive coronary artery disease (CAD). Among obstructive CAD cases, PCI was performed in 77% and CABG in 21% of cases; 26% had a repeat procedure and 11% died from non-cardiac causes. When comparing PWH who had CC to those who did not, there was a significantly higher rate of statin use (63% vs. 25%, *p* < 0.015) and a higher prevalence of low ejection fraction (38% vs. 11%, *p* = 0.004) among those patients who underwent CC. However, there was no significant difference in the prevalence of hypertension (*p* = 0.13), HbA1c levels (*p* = 0.32), CD4 count (*p* = 0.45) nor in undetectable viral load status (*p* = 0.75) after controlling for age, sex and BMI. Despite the finding of traditional CVD risk factors among PWH, there were no differences in HIV-related factors among patients requiring CC, supporting the importance of optimization of traditional CVD risk factors in this population.

## 1. Introduction

In recent years, both the efficacy and availability of antiretroviral medication therapy for human immunodeficiency virus (HIV) infection has improved dramatically. Consequently, the past two decades have seen an increase in life expectancy of persons living with HIV (PWH) [1]. These changes have led to a decrease in HIV-related conditions, but a rise in non-AIDS-related comorbidities, most notably cardiovascular diseases (CVD) [2].

The pathophysiology of CVD in the context of HIV is multifactorial and not yet fully understood. However, it is thought that an important contributing factor is the increase in inflammatory and prothrombotic states in PWH, which can lead to increased endothelial dysfunction and coagulation abnormalities, with subsequent CVD manifestation at an earlier onset in this population [3,4,5]. Indeed, patients with long-standing HIV have been shown to have a higher prevalence of coronary artery stenosis, with plaques positive for protein p24, an HIV protein [6]. In addition, the HIV virus affects macrophage differentiation contributing to the formation of vulnerable plaques, which have a higher risk of rupture and may lead to an acute coronary syndrome (ACS) [7,8]. Investigations suggest that PWH with just one risk factor for coronary artery disease (CAD) may have a two-fold increased risk of acute myocardial infarction (MI) [9]. Furthermore, this increased risk rises to 3.6-fold in PWH with three or more CAD risk factors, compared to HIV-seronegative patients [10]. In addition, PWH with acute MI carry a 4.6-fold increased risk of repeat MI at one year follow-up, with a 4.5-fold increased risk for sudden cardiac death compared to the general population [11,12].

The presence of HIV seropositivity is associated with a 61% increased risk of CAD, with women at a higher risk independent of age [2,9,10]. However, in the previous two decades, patients with HIV were less likely to receive invasive management with cardiac catheterization (CC), and less likely to have drug-eluting stents (DES) placed compared to the general population [13,14].

Florida is the state with the highest rate of new HIV infections in the United State (US), per the Center for Disease Control report [15]. Due to the increase in the prevalence of CVD morbidity and mortality in PWH, we sought to examine the current status of CC procedures among PWH in South Florida.

## 2. Materials and Methods

This study was a retrospective chart review approved by the Institutional Review Board (IRB) at the University of Miami Miller School of Medicine (approval number 20161109).

### 2.1. Subjects and Location

A retrospective chart review of 985 patients evaluated at the Special Immunology clinics between January 2017 and November 2019 was performed. This time frame allows us to have the most recent data for analysis. Patients included were not limited by payor status (Medicare, Medicaid, private, self-pay, or other insurance). No patients under the age of 18 were included in this study.

### 2.2. Procedures

Patients were randomly selected to minimize sampling bias during data collection. A retrospective chart review was performed on the selected patients using the Electronic Medical Record (EMR), examining clinic notes, anthropometric data, laboratory tests, diagnostic tests, and outcomes. 

Clinic notes were reviewed to identify diagnoses, relevant comorbidities, HIV-related medications, and lifestyle behaviors. Anthropometric data was reviewed for blood pressure (BP) and body mass index (BMI). Review of laboratory tests included markers for cardiovascular risk and HIV-related markers, such as CD4 counts and viral load. Diagnostic testing for cardiovascular disease that were on file was reviewed, including echocardiographic data, stress testing and cardiac catheterization reports. Primary outcomes were cardiovascular comorbidities and HIV-related factors. Secondary outcomes included the need for repeat CC, coronary artery bypass graft (CABG) and/or death.

### 2.3. Statistical Analysis

All individuals who underwent CC were identified. The indication for CC was obtained from the cardiac catheterization report, such as echocardiogram or stress test findings, or acute coronary syndrome at presentation. Furthermore, we reviewed the CC reports to determine the findings such as the culprit artery, number of vessels affected, and type of stent used.

To estimate the most recent nationwide prevalence of CC, PCI and repeat PCI in the US, we used weighted discharge data from the National Inpatient Sample (NIS) during 2017. International Classification of Diseases, Tenth Revision, Procedure Coding System (ICD-10-PCS) (see Table S1 for ICD-10-PCS codes).

We performed a comparison of clinically relevant characteristics between PWH who underwent CC, and PWH not undergoing CC. To identify statistically significant differences in variables between both groups, *t*-test for parametric variables or Mann–Whitney U tests for non-parametric variables were used. The Pearson Chi Squared test was used to evaluate the differences between categorical variables. Multivariate logistic models were used as the binary outcome to identify possible predictors of CC. Variables were added in a stepwise manner based on clinical judgement, and known and hypothesized relationships between HIV and CVD.

## 3. Results

### 3.1. Baseline Characteristics of the Population

Among 985 PWH, 538 (54.6%) were males. The age of patients ranged from 22 to 88 years with an average of 52.2 ± 11.7 years, and a median of 53.0 years. The majority of subjects self-identified as African American (AA)/Black (65.4%), while 33.4% identified as White, and 1% identified as other races. In the entire cohort, 18.5% reported current alcohol use, 23.9% were smokers (current or past), and 7.8% were drug users (current or past). The mean systolic blood pressure (SBP) was 129 mmHg and diastolic blood pressure (DBP) was 74 mmHg, and body-mass index mean was 28.68 ± 6.86. Among laboratory data, mean total cholesterol was 182 mg/dL, HDL of 49 mg/dL, LDL average was 105 mg/dL, and average glycohemoglobin (HbA1c) of 5.96%. With regard to medical therapy, 92.9% of PWH were on ART, with 24.2% prescribed a PI, with an average CD4 count of 568 cells/mm. Overall, 71.2% of patients achieved an undetectable viral load (See Table 1).

### 3.2. Comparison of PWH Undergoing or Not Cardiac Catheterization

The total prevalence of PWH undergoing CC in our cohort was 1.9% (n = 19), specifically 0.8% in males (n = 8) and 1.1% in females (n = 11). Primary indications for CC were abnormal stress echocardiogram (47.4%), followed by acute coronary syndrome (31.6%), stable angina (15.7%), and abnormal two-dimensional transthoracic echocardiogram (5.3%). In patients undergoing CC, 32% were found to have non-obstructive CAD compared to 68% with obstructive CAD. Among patients with obstructive CAD lesions, most had single-vessel disease (53.8%), compared to two-vessel disease (23.1%) or triple-vessel disease (15.4%). The most common culprit vessels affected during the first CC were the left anterior descending artery (LAD, 50%) followed by the right coronary artery (RCA, 30.0%). 

Amongst CC procedures that documented obstructive CAD, percutaneous coronary intervention (PCI) was performed in 77% of cases, and CABG in 21% of cases immediately after CC. All patients with triple-vessel CAD underwent CABG. Among catheterizations requiring PCI, drug-eluting stents (DES) were deployed in 67% of cases compared to bare metal stents (BMS) in 33% of cases. 

Interestingly, among PWH requiring CC, 26% had a history of repeat intervention, ranging from 2 weeks to 4 years after the initial CC procedure. In one case, repeat CC was performed for in-stent restenosis, while two patients had more than two CC during the period of time previously mentioned. After repeat CC, the LAD was found to be affected in 40% of the cases, RCA in 27%, and left circumflex artery (LCx) in 20% of the cases. We found that 10.5% of PWH who underwent CC died from non-cardiac causes (motor vehicle accidents in all cases).

### 3.3. Predictors of Cardiac Catheterization Procedures

Patients undergoing CC were older when compared to those PWH that did not undergo CC (average 60.21 ± 8.61 years vs. 52.04 ± 11.73 years, *p* = 0.003). While there was a larger proportion of males and AA/Black in the group undergoing CC, these differences were not statistically significant (*p* = 0.77 and *p* = 0.09, respectively). No significant differences were noted between groups regarding systolic and diastolic blood pressures (126 ± 26 mmHg vs. 132 ± 21 mmHg, *p* = 0.48; and 70 ± 12 mmHg vs. 75 ± 12 mmHg, *p* = 0.14, respectively), however, there was a significantly higher prevalence of HTN in patients requiring CC (73.7% vs. 57.1%, *p* = 0.007). No significant difference was noted between groups for reported tobacco, alcohol or illicit drug use.

We found a significantly lower level of total cholesterol (*p* = 0.037) and LDL cholesterol level (*p* = 0.046) in PWH undergoing CC. No differences were noted in HDL cholesterol or triglyceride levels between groups. However, statin therapy prescription rates were significantly higher among patients that underwent CC (63.2% vs. 24.9%, *p* < 0.05). Similarly, mean hemoglobin A1c was significantly higher among PWH that underwent cardiac catheterization (6.42 ± 1.35% vs. 5.95 ± 1.39%, *p* = 0.034).

In regard to viremic control, no significant differences in mean CD4 counts or viral load undetectability were noted between groups (523 ± 319 vs. 569 ± 338 cells/mm^3^, *p* = 0.64, and 78.9% vs. 71%, *p* = 0.45, respectively). Similarly, ART prescription rates were comparable between groups (100% vs. 92.8%; *p* = 0.22). Specifically, protease inhibitor use was also comparable between groups (26.3% vs. 24.2%; *p* = 0.82). 

Among all PWH in our cohort, 265 patients underwent a transthoracic echocardiogram. Of these, a significantly higher proportion of abnormal echocardiograms were performed in patients undergoing CC (75% vs. (14.5%, *p* < 0.05). An abnormal echocardiogram was considered when individuals had a low ejection fraction, wall motion abnormalities, valvular heart disease, or pericardial effusion. Conversely, PWH that did not have documented CC had a significantly greater proportion of preserved left ventricular ejection fraction above or equal to 55% (88.8% vs. 62.5%, *p* = 0.002) (See Table 1).

A logistic regression of PWH that underwent CC as the outcome, to identify predictors, was performed. Age remained a consistently significant predictor in all models, with 6.9% higher odds of undergoing CC for every year of life (r^2^ = 0.010, OR 1.069, *p* = 0.003), even after controlling for other variables. Gender, BMI, smoking history, HTN, DM, undetectable VL, CD4 count and ART medications were not significant predictors in our models (see Table 2).

## 4. Discussion

In this study, we identified within a cohort of PWH a 1.9% prevalence of cardiac catheterization. This represents a higher prevalence than that previously reported in PWH and is also higher than what is reported from the public data on the general population for any age. Although it is difficult to determine the precise number of CC in the US, using an estimate with the NIS database from 2017, there was an average of 1,026,620 CC in the US with a population of 325.1 million in the same year, corresponding to a prevalence of 0.3% CC in the US during that year. Similarly, there were approximately 481,865 PCIs, which represents 47% of individuals who underwent CC. A similar finding was seen per the last update from American Heart Association in 2019, the estimated prevalence of CC in the general population was 0.3%, of which 47% of patients undergoing CC required PCI [16]. Previous studies have suggested that PWH may undergo less frequent invasive procedures after ACS compared to seronegative populations [17]. However, this may be center dependent, as others studies have shown little to no treatment bias in selection of PWH for coronary angiography relative to the general population [18].

In this study, we found an increased rate of women undergoing CC that goes along with previous studies that showed an excess risk of cardiovascular events in PWH that is amplified in women [2,19,20]. Prior investigations revealed that PWH have increased traditional risk factors for CVD, such as HTN, DM, or obesity, among others [2]. In our cohort, PWH requiring CC did not have significantly increased prevalence of a number of traditional risk factors (systolic and diastolic blood pressure, BMI, reported tobacco, alcohol, illicit substance use). However, a larger proportion of PWH undergoing CC carried a diagnosis of HTN. Further, PWH undergoing CC had a lower total cholesterol and LDL cholesterol, with no difference in HDL cholesterol and triglycerides levels, likely due to a demonstrated accompanying increase in statin therapy among these patients. Per previous studies, the presence of dyslipidemia has been present when evaluating the association between HIV and CVD [21]. Overall, a degree of uncertainty exists in assessing the proportion of statin use that preceded, rather than followed, cardiac catheterization given the reliance on prescription history through electronic medical record review.

In terms of glycemic control, PWH undergoing CC had significantly higher HbA1c levels compared to those that did not have a CC. No differences were noted in the diagnoses of type I or II DM, although abnormal glycemic control and metabolic syndrome among these patients may certainly increase the risk of CVD [22].

Past studies have reported that women living with HIV have a significantly higher risk of CVD compared to seronegative females, and are less likely to be re-vascularized by PCI [23,24,25]. However, in this study, we found a high prevalence of women with HIV that underwent CC (41.2%) and PCI (36.4%). Indeed, the percent of women requiring CC was higher than men, highlighting the importance of gender differences in CVD manifestations.

On another hand, viremic control and accompanying leukocyte count have been demonstrated to play an important role in CVD risk among PWH. Lower CD4 counts have been independently associated with higher rates of MI, particularly when counts dropped below 500 cells/µL [26]. In our study, the majority of the patients had CD4 counts > 500 and there was no statistically significant difference in the median CD4 counts between groups. CD4 count was also not a statistically significant predictor of need for CC in the multivariate model, after controlling for demographic and lifestyle factors (see Table 2). Although a previous study demonstrated that a lower nadir CD4 count was associated with a greater likelihood of PCI, in this population where HIV factors were mostly controlled, we did not find any significant association among HIV-related factors and CC [27].

Successful ART results in undetectable blood viral load in persons seropositive for HIV, as measured by existing clinically approved assays [28]. However, chronic HIV infection is associated with persistent immune activation, a pro-inflammatory state, and an increased risk of non-AIDS chronic diseases [29,30]. In previous studies, PWH with detectable viral loads displayed significantly higher risks for comorbidities or clinical markers of comorbidities, suggesting a link between HIV viremia and comorbidity [31,32]. However, detailed analyses of this association are hampered by the fact that the majority of PWH in our cohort have an undetectable viral load, supporting the stability of HIV-related factors.

Guidelines recommend a stepwise approach when evaluating for CAD. When there are symptoms suggestive of CAD, non-invasive studies including stress tests are performed. If noninvasive test results are abnormal, consideration is done to undergo further evaluation of coronary anatomy, usually with a CC [33,34]. In our study, indication for CC was mainly abnormal stress echocardiography (47.4%). The proportion of abnormal testing that we found was relatively lower when compared to a study from the general population (71%); however, in that study ACS was excluded, while in our cohort ACS was part of the indications for CC [23]. Increased awareness of CVD risk among PWH has likely resulted in increased frequency of cardiac catheterization, compared to prior reports. Increased pro-inflammatory states may lead to plaque rupture and heightened risk of ACS in this population [35]. However, in ageing populations of PWH as in this cohort, we also found that a significant percentage of CC were done due to abnormal stress tests, supporting increased incidence of stable CAD among PWH at a mean age of 60 years.

PWH undergoing CC were found to have single vessel disease in 53.8% of the cases. This finding is similar to previous studies showing a higher proportion of single vessel disease in PWH when compared to the general population (ranging from 20 to 40%) [36,37,38]. It was also shown that PWH have fewer complex lesions, but more vulnerable plaques than the general population, suggesting different pathophysiologic processes in PWH [25,39]. We found a higher proportion of repeat procedures in our cohort (26%) when compared to what is reported in the NIS database from 2017, where in the general population individuals with a history of previous PCI, 19.3% had a repeat PCI.

Last, among PWH requiring PCI in this cohort, deployed stents were most commonly drug-eluting. In the past, PWH were less likely to receive DES and more likely to receive BMS. However, recent studies along with these data support that this is no longer the case among PWH [40,41].

### Limitations

Due to the small number of PWH that underwent CC compared to the group not undergoing CC, the differences found in this study might be related to other possible confounding factors not taken into account that preclude a full comparison between both groups. Sampling bias may occur as the patients included were seen only within a dedicated HIV clinic. This was a cross sectional, not a longitudinal study; therefore, no data on changes of risk factor profiles over time, duration of exposure to ARTs, duration of exposure to HIV, post discharge adherence to medications including DAPT therapy, outpatient follow up and cardiac rehabilitation at discharge could be assessed. Recall bias may also affect patient reported rates of tobacco, alcohol, and drug use. Regarding both statin therapy and ART prescription onset/duration, we were unable to identify whether the medication was started before or after CC. We were unable to generalize the results of this study relative to the rest of the region or country, as this study was performed at a single academic center with the majority of patients being AA and Hispanics. In addition, the lack of seronegative individuals in our cohort allows for limited comparison.

## 5. Conclusions

PWH, despite mostly having an undetectable VL and controlled CD4 count, are at higher risk of cardiovascular disease. This is now being reflected in the higher prevalence of CC being performed in this population, particularly among women. These data support the need for further interventions to improve primary and secondary cardiovascular disease prevention in this aging population.

## Figures and Tables

**Table 1:** General data of the entire cohort and comparison of patients undergoing CC vs. non-CC group.

Covariables (Units)	PWH Cohort (N = 985)	CC (N = 19; 1.9%)	NO CC (N = 966; 98.1%)	*p* Value
**AGE (YEARS)**	52.20 ± 11.73	60.21 ± 8.61	52.04 ± 11.73	**0.003**
**SEX**				
**FEMALE**	447 (45.4%)	8 (42.1%)	439 (45.4%)	0.772
**MALE**	538 (54.6%)	11 (57.9%)	527 (54.6%)	
**RACE**				
**WHITE**	331 (33.6%)	2 (10.5%)	329 (34.1%)	**0.027**
**AA/BLACK**	644 (65.4%)	16 (84.2%)	628 (65%)	0.089
**OTHERS**	10 (1.0%)	1 (5.3%)	9 (0.9%)	0.057
**ETHNICITY HISPANIC**	329 (33.4%)	3 (15.8%)	326 (33.7%)	0.100
**HAITIAN**	110 (11.2%)	1 (5.3%)	109 (11.3%)	0.409
**SBP (MMHG)**	132 ± 21	129 ± 26	132 ± 21	0.478
**SBP (MMHG)**	75 ± 12	70 ± 12	75 ± 12	0.136
**HTN**	428 (43.5%)	14 (73.7%)	552 (57.1%)	**0.007**
**BMI (KG/M2)**	28.68 ± 6.86	29.07 ± 7.88	28.67 ± 6.84	0.937
**TOTAL CHOLESTEROL (MG/DL)**	182 ± 43	161 ± 44	183 ± 43	**0.037**
**HDL (MG/DL)**	49 ± 18	44 ± 15	49 ± 18	0.130
**LDL (MG/DL)**	105 ± 42	86 ±3 8	106 ± 42	**0.046**
**TRIGLYCERIDES (MG/DL)**	149 ± 97	158 ± 92	149 ± 97	0.547
**STATIN USE**	253 (25.7%)	12 (63.2%)	241 (24.9%)	**<0.05**
**HBA1C (%)**	5.96 ± 1.40	6.42 ± 1.35	5.95 ± 1.39	**0.034**
**VIRAL LOAD (COPIES/ML)**	8283 ± 58,853	25 ± 86	8450 ± 59,434	0.302
**UNDETECTABLE VL**	682 (71.2%)	15 (78.9%)	667 (71%)	0.451
**CD4 COUNT (CELLS/MM3)**	568 ± 337	523 ± 319	569 ± 338	0.637
**ON ART**	915 (92.9%)	19 (100%)	896 (92.8%)	0.223
**ON ANY PI**	238 (24.2%)	5 (26.3%)	233 (24.1%)	0.825
**CURRENT SMOKER**	235 (23.9%)	1 (5.3%)	234 (24.2%)	0.055
**CURRENT ALCOHOL USE**	182 (18.5%)	3 (15.8%)	179 (18.5%)	0.761
**CURRENT DRUG USER**	77 (7.8%)	0 (0%)	77 (8.0%)	0.200
**ABNORMAL ECHO**	152 (15.4%)	12 (63.2%)	140 (14.5%)	**<0.05**
**EF < 55%**	34 (3.5%)	6 (37.5%)	28 (11.2%)	**0.002**
**ABNORMAL STRESS TEST**	12 (1.2%)	5 (26.3%)	7 (0.7%)	**<0.05**

PWH = Person living with HIV; AA = African American; SBP = Systolic blood pressure; DBP = Diastolic blood pressure; HTN = Hypertension; BMI = Body Mass Index; HbA1c = Glycohemoglobin; VL = Viral load; ART = Antiretroviral therapy; PI = Protease inhibitors; Echo = echocardiogram; EF = Ejection fraction. Bold = significant difference with *p* < 0.05 as significant.

**Table 2:** Multivariate logistic models to identify possible predictors of CC.

	Model 1		Model 2		Model 3		Model 4		Model 5		Model 6		Model 7		Model 8	
**Observations**	985		966		966		966		966		943		935		966	
**Cox and Snell R Square**	0.010		0.010		0.13		0.13		0.18		0.21		0.19		0.20	
	OR	SE	OR	SE	OR	SE	OR	SE	OR	SE	OR	SE	OR	SE	OR	SE
**Age**	1.069 *	0.022	1.071 *	0.023	1.059 *	0.025	1.056 *	0.025	1.053 *	0.025	1.053 *	0.025	1.053 *	0.025	1.054 *	0.025
**Gender**	1.095	0.472	1.169	0.479	1.211	0.481	1.206	0.480	1.278	0.481	1.325	0.485	1.196	0.485	1.307	0.482
**BMI**	-	-	1.023	0.034	1.014	0.037	1.011	0.036	1.005	0.037	1.003	0.037	1.014	0.037	1.003	0.037
**HTN**	-	-	-	-	2.315	0.565	2.233	0.560	2.182	0.565	2.102	0.567	2.264	0.566	2.160	0.566
**DM**	-	-	-	-	-	-	1.367	0.525	1.315	0.527	1.307	0.528	1.359	0.529	1.266	0.529
**Smoking**	-	-	-	-	-	-	-	-	0.185	1.035	0.196	1.036	0.174	1.036	0.193	1.036
**Viral load**	-	-	-	-	-	-	-	-	-	-	0.999	0.001	-	-	-	-
**CD4 Count**	-	-	-	-	-	-	-	-	-	-	-	-	0.999	0.001	-	-
On ART	-	-	-	-	-	-	-	-	-	-	-	-	-	-	0	4976

BMI = Body Mass Index; HTN = Hypertension; DM = Diabetes mellitus; ART = Antiretroviral therapy. Variables were added in a stepwise manner based on clinical judgement. * significant finding with *p* < 0.05.

## Data Availability

The data that support the findings in this study are available from corresponding author, B.E., upon reasonable request.

## Supplementary Materials

The following are available online at https://www.mdpi.com/article/10.3390/jcdd8040033/s1, Table S1: ICD-10 codes used.
