# Supplementary material for: Cardiac Catheterization Procedures in Patients with HIV: A Retrospective Analysis
Source: J Cardiovasc Dev Dis. 2021 Mar 27;8(4):33. doi: 10.3390/jcdd8040033 (PMC8066790; doi:10.3390/jcdd8040033)
Supplement: Supplementary file 1 [file jcdd-08-00033-s001.pdf]

SUPPLEMENTAL MATERIAL

Data Source

We utilized the NIS databases from 2017, which is a database developed by HCUP, a Federal-State-Industry partnership sponsored by the Agency for Healthcare Research and Quality (AHRQ). It contains data on inpatient hospital stays from all participating states (n=47 plus the District of Columbia) in 2017. The NIS is the largest, all-payer inclusive inpatient database that is publicly available, and it contains data on approximately 7 million discharges annually from more than 1000 hospitals, to approximate a 20% stratified and weighted sample of inpatient discharges from all US community hospitals (defined as all non-federal, short-term, general and other subspecialty hospitals, excluding rehabilitation and long-term acute care hospitals). The NIS is representative of more than 97% of the US population. Inpatient stay records in the NIS are composed of clinical and other resource utilization information available from discharge abstracts derived from state-mandated discharge reports [1]. Discharge weights provided with the NIS files can be used to derive national estimates [2]. From 2017, a total of 7,159,352 discharge records comprised the NIS for the US adult population (age ≥ 18), corresponding to a national estimate of 35,796,743 hospitalizations across the country. We identified all patients aged ≥ 18 undergoing cardiac catheterization and percutaneous coronary intervention using ICD-10-PCS code specified in Supplemental Table 1 in any procedure field.

References:

1. HCUP National Inpatient Sample (NIS). Healthcare Cost and Utilization Project (HCUP). Agency for Healthcare Research and Quality, Rockville, MD. www.hcupus.ahrq.gov/nisoverview.jsp. Accessed May 9, 2020.
2. Trend Weights for HCUP NIS Data. http://www.hcupus.ahrq.gov/db/nation/nis/trendwghts.jsp. Accessed May 9, 2020.

Table S1: ICD-10 codes used.

Variables	ICD-10-PCS
Cardiac Catheterization	B210010, B2100ZZ, B210110, B2101ZZ, B210Y10, B210YZZ, B211010, B2110ZZ, B211110, B2111ZZ, B211Y10, B211YZZ, B212010, B2120ZZ, B212110, B2121ZZ, B212Y10, B212YZZ, B213010, B2130ZZ, B213110, B2131ZZ, B213Y10, B213YZZ, B2150ZZ, B2151ZZ, B215YZZ, B2160ZZ, B2161ZZ, B216YZZ, B2170ZZ, B2171ZZ, B217YZZ, B2180ZZ, B2181ZZ, B218YZZ, B21F0ZZ, B21F1ZZ, B21FYZZ
Percutaneous Coronary Intervention	02703ZZ, 02704ZZ, 02713ZZ, 02714ZZ, 02723ZZ, 02724ZZ, 02733ZZ, 02734ZZ, 02Q03ZZ, 02Q04ZZ, 02Q13ZZ, 02Q14ZZ, 02Q23ZZ, 02Q24ZZ, 02Q33ZZ, 02Q34ZZ, 0270346, 027034Z, 0270356, 027035Z, 0270366, 027036Z, 0270376, 027037Z, 02703D6, 02703DZ, 02703E6, 02703EZ, 02703F6, 02703FZ, 02703G6, 02703GZ, 02703T6, 02703TZ, 02703Z6, 0270446, 027044Z, 0270456, 027045Z, 0270466, 027046Z, 0270476, 027047Z, 02704D6, 02704DZ, 02704E6, 02704EZ, 02704F6, 02704FZ, 02704G6, 02704GZ, 02704T6, 02704TZ, 02704Z6, 0271346, 027134Z, 0271356, 027135Z, 0271366, 027136Z, 0271376, 027137Z, 02713D6, 02713DZ, 02713E6, 02713EZ, 02713F6, 02713FZ, 02713G6, 02713GZ, 02713T6, 02713TZ, 02713Z6, 0271446, 027144Z, 0271456, 027145Z, 0271466, 027146Z, 0271476, 027147Z, 02714D6, 02714DZ, 02714E6, 02714EZ, 02714F6, 02714FZ, 02714G6, 02714GZ, 02714T6, 02714TZ, 02714Z6, 02714ZZ, 0272346, 027234Z, 0272356, 027235Z, 0272366, 027236Z, 0272376, 027237Z, 02723D6, 02723DZ, 02723E6, 02723EZ, 02723F6, 02723FZ, 02723G6, 02723GZ, 02723T6, 02723TZ, 02723Z6, 0272446, 027244Z, 0272456, 027245Z, 0272466, 027246Z, 0272476, 027247Z, 02724D6, 02724DZ, 02724E6, 02724EZ, 02724F6, 02724FZ, 02724G6, 02724GZ, 02724T6, 02724TZ, 02724Z6, 0273346, 027334Z, 0273356, 027335Z, 0273366, 027336Z, 0273376, 027337Z, 02733D6, 02733DZ, 02733E6, 02733EZ, 02733F6, 02733FZ, 02733G6, 02733GZ, 02733T6, 02733TZ, 02733Z6,

	02733ZZ, 0273446, 027344Z, 0273456, 027345Z, 0273466, 027346Z, 0273476, 027347Z, 02734D6, 02734DZ, 02734E6, 02734EZ, 02734F6, 02734FZ, 02734G6, 02734GZ, 02734T6, 02734TZ, 02734Z6, 02734ZZ
History of previous Percutaneous Coronary Intervention	Z98.61, Z95.5
